# Design and adjustment of the graphene work function via size, modification, defects, and doping: a first-principle theory study

**DOI:** 10.1186/s11671-017-2375-3

**Published:** 2017-12-29

**Authors:** Ning Yang, Daoguo Yang, Liangbiao Chen, Dongjing Liu, Miao Cai, Xuejun Fan

**Affiliations:** 1 The Faculty of Mechanical and Electrical Engineering, Guilin University of Electronic Technology, Guilin, 541004 China; 2 The Department of Mechanical Engineering, Lamar University, Beaumont, 77706 USA

**Keywords:** Work function, Graphene, Functional groups, Defects, Positions, Doping, First-principle theory

## Abstract

In this work, the work function (WF) of graphenes, which are used as electronic devices, has been designed and evaluated by using the first-principle approach. Different states of graphene were considered, such as surface modification, doping, and defects. Firstly, WF strongly depends on the width of pristine graphene. A bigger width leads to a smaller WF. In addition, the effects of hydroxyls, defects, and positions of hydroxyls and defects are of concern. The WF of the graphene which is modified with hydroxyls is bigger than that of the pristine graphene. Moreover, the WF value increases with the number of hydroxyls. Positions of the hydroxyls and defects that deviated from the center have limited influence on the WF, whereas the effect of the position in the center is substantial. Lastly, B, N, Al, Si, and P are chosen as the doping elements. The n-type graphene doped with N and P atoms results in a huge decline in the WF, whereas the p-type graphene doped with B and Al atoms causes a great increase in the WF. However, the doping of Al in graphene is difficult, whereas the doping of B and N is easier. These discoveries will provide heavy support for the production of graphene-based devices.

## Background

As a material that possesses a variety of excellent performances, graphene [1–3] has been widely used in different areas, such as sensors, field-effect transistors (FET), electrode of photovoltaic devices, Schottky diodes, vacuum tube, and metal–semiconductor junction of light-emitting diodes, and has become a substitute for many materials [4–7]. Graphenes can solve miniaturization problems of FET and the cost of photovoltaic devices while maintaining good stability and electrical performance. However, graphene work function (WF) has a crucial influence on the performance of these electronic devices. Therefore, knowing and controlling the WF of graphenes is of great significance to graphene-based electronic devices. Generally, the performance of FET devices can be determined by the WF of source/drain electrodes [8–10]. With the differences in WF of materials after the metal–semiconductor contact, a potential difference will exist in the interface, which has a direct effect on Schottky or ohmic contact [10]. Given that the band alignment of two different materials is determined by their respective WFs, controlling the graphene WF is the key in reducing the contact barriers [11].

Graphene WF measured via experiment is approximately at 4.2 to 4.8 eV [12, 13]. The change of Fermi level will lead to the change of WF. Many experiments and theoretical analysis showed that the Fermi level of graphenes can be adjusted through deliberate doping by aromatic and gas molecules [14, 15] or ultraviolet irradiation [16], surface functionalization [17, 18], defects [19], and electrostatic gating [20]. For example, Yuan et al. found that the WFs of graphene change dramatically via the adsorption of Na and Cl [21]. Zhang et al. showed that the WF can be finely tuned within the range of 4.0–4.5 eV by covering the graphene with alkali metal cations [22]. Leenaerts et al. learned the graphene intrinsic characteristics. The results showed that the WF of few­layer graphene was almost independent of the number of layers, but it can be modulated by dipole layer [23]. Volodin et al. and Peng et al. used the mechanical method to change the graphene WF [24]. All of them found that the WF will increase with the strain. Yu et al. used electric field effects to adjust the WF of graphene and demonstrated that the WF can be tuned within the range 4.5–4.8 eV for monolayer graphenes and 4.65–4.75 eV for bilayer graphenes in ambient and dry nitrogen conditions [25]. Shi et al. found that the surface potential of graphene films can be adjusted by controlling the immersion time. For doping time less than 20 s, the surface potential was monotonically increased to about 0.5 V [13]. Moreover, irradiation was found to be an efficient method in controlling the doping concentration. Stratakis et al. controlled the doping and reaction levels to tailor the WF of the GO–Cl layers from 4.9 eV to a maximum value of 5.23 eV by tuning the laser exposure time [26]. However, Kang et al. tuned the WF of graphene oxide via direct surface functionalization [27].

Although many previous studies have reported methods to control graphene WF, the research results are not comprehensive enough. For example, the comparative study about the size effect of different chiral graphene on WF does not provide sufficient information. Additionally, the effects of graphene’s modifications and defects on WF are still not very clear. Although the effect of doping on graphene WF was studied, the corresponding formation energy of doping atoms was not mentioned. For example, in Shi’s experiment, the graphene was immersed in an AuCl_3_ solution to adjust the WF [13]; however, the relationship between WF and doping concentration was still unclear. In addition, it has to be noted that the impacts of the positions of functional groups and defects on graphene WF have not yet been reported. Given the expensive cost of WF’s controlling methods, intrinsic characteristics of the different methods must be investigated.

In this paper, a comprehensive study on the controlling methods of the WF was investigated via the first-principle theory. Effects of the doping and the positions of hydroxyls and defects were first reported and highlighted. First, graphenes with different chirality (zigzag and armchair) were considered, and the dependence of WF on the graphene width investigated. Second, the WFs of the graphene with surface modifications and defects were calculated. Different distributions of hydroxyls were first compared, followed by the effect of defects at various positions. Third, B, N, Al, Si, and P were chosen as the doping elements to study the doping effect of WFs.

## Methods

All calculations were performed in CASTEP code based on the density functional theory (DFT) [28], which is a kind of quantum mechanics research for the electronic structure of the multi-electron system. DFT has been widely used in the study of physical and chemical properties, including nanomaterials of graphenes and carbon nanotubes [29, 30]. DFT can also accurately simulate tens to hundreds of atomic systems and describe the atom as quantum particles, namely, the set of nuclei and electrons [31].

The generalized gradient approximation (GGA) and local density approximation (LDA) are the exchange–correlation functionals commonly used in quantum mechanics calculations. They are described in Eqs. (1) and (2):

1 $$ {E}_{\mathrm{xc}}\ \left[\rho \right]=\int {f}_{\mathrm{xc}}\left[\ \rho \left(\boldsymbol{r}\right),|\Delta  \rho \left(\boldsymbol{r}\right)\ |\right]d\boldsymbol{r} $$

2 $$ {E}_{\mathrm{xc}}\ \left[\rho \right]=\int d\boldsymbol{r}\ \rho \left(\boldsymbol{r}\right)\ {\varepsilon}_{\mathrm{xc}}\ \left[\rho \left(\boldsymbol{r}\right)\right] $$

where ***R*I** and ***r*** are the coordinates of the atomic nucleus and the electron, respectively. The exchange–correlation energy in inhomogeneous electron gas is replaced by the *E*_xc_[*ρ*] in uniform electron gas. Both GGA and LDA have been used for the calculations in two-dimensional materials. Lebègue et al. found that the band structure of two-dimensional materials obtained using either LDA or GGA is very similar [32]. At the same time, GGA was used in the calculation of the electric properties of graphene in Kharche’s and Gui’s researches, which guarantees the accuracy [33, 34].

As for the WF, the previous scanning probe-based studies had shown that the WF is measured as 4.6 eV, such as with graphite [35]. Generally, WFs in the range of 4.6–4.9 eV are acceptable [36, 37]. In addition, the WF was predicted by LDA [38] and GGA [39] as 4.48 and 4.49 eV, respectively. In comparison with the experiment date, the WF calculated by theory is slightly smaller. GGA has joined a non-local density gradient and its nonlocality is more suitable for processing the inhomogeneity of density, but LDA works better in a stacking system. Therefore, in the calculations of WF and electric property of graphene, GGA was chosen in this theoretical study. Furthermore, in this calculation, the vacuum distance is set as 15 Å so that the electrostatic interactions between two sides of a slab are negligible, and the electrostatic potential reaches its asymptotic value. The ultrasoft pseudopotential is used to describe the interaction between electrons and ions. Cutoff energy is at 340 eV, the Brillouin zone is sampled using a 9 × 9 × 1 Monkhorst–Pack k-point grid [40], and Methfessel–Paxton [41] smearing is at 0.05 eV. The convergence criterion of self-consistent field energy was 1.0 × 10^−6^ eV, and the MAX force is 0.03 eV/Å.

## Results and discussion

### WF of zigzag and armchair graphenes with different sizes

Generally speaking, WF can be defined as the minimum energy needed to extract an electron from bulk to infinity [42]. As in quantum mechanics calculations, WF is defined as the difference between the vacuum level (*V*_0_) and the Fermi level (*E*_f_), as shown in Eq. (3):

3 $$ \mathrm{WF}={V}_0-{E}_{\mathrm{f}} $$

**Fig. 1:**
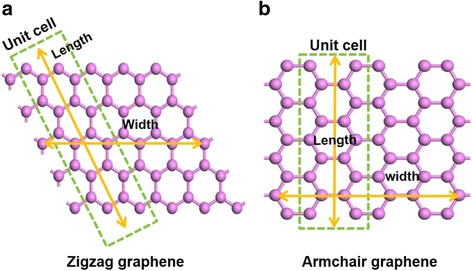
The definition of the graphene’s size. The schematic structures of zigzag (**a**) and armchair (**b**) graphenes that illustrate the definition of the graphene’s size. A unit cell is set as a carbon ring in the green box. The yellow arrows represent the direction of width and length

CASTEP calculations for crystal surfaces are carried out on slabs with a region of vacuum. Effectively, an infinite array of 2D-periodic slabs of material is separated by wide vacuum spacings. CASTEP produces the Fermi energy for such systems and the spatial distribution of the electrostatic potential [43]. Graphene with different widths has various properties. The models with different chirality of zigzag and armchair were chosen to elucidate the effect of width on the WF. In this calculation, samples with the range of one- to seven-unit cells were calculated. Figure 1 illustrates the definition of the size of zigzag and armchair graphenes. The crystal orientation of zigzag and armchair graphenes is different; the crystal structure of zigzag graphene is rhombic but the crystal structure of armchair graphene is dimetric, as shown in Fig. 1a, b. The width of the graphene is defined in the horizontal direction, and the length of the graphene is defined in the vertical direction. In addition, a unit cell is set as a carbon ring.

**Fig. 2:**
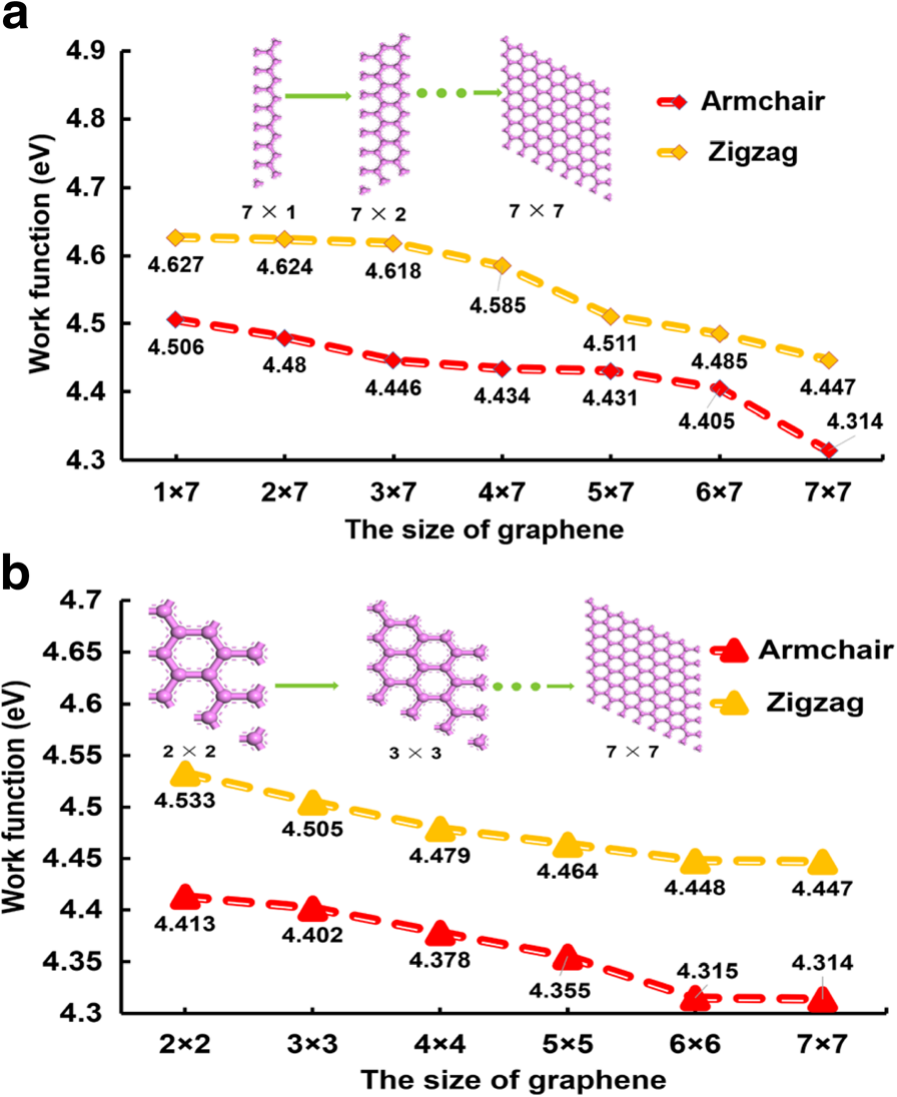
The relationship between graphene size and WF. The relationship between graphene size and WF. The length and width of graphenes are different in (**a**), whereas the same in (**b**)

Graphene band gap changes with the change of graphene’s width. Generally speaking, the zigzag graphene presents a metallic property, and armchair graphene shows a half-metallic property. However, what is the relationship between WF and width in graphenes? Figure 2 shows the relationship between the graphene size and the WF. The length and width of graphenes are unequal in Fig. 2a in which the length is constantly set as seven-unit cells but the width is arranged from one-unit to seven-unit cells (1 × 7 to 7 × 7), whereas the length and width are equal in Fig. 2b in which the size is arranged from 2 × 2 supercells to 7 × 7 supercells. The WF is affected greatly by the graphene width. Generally, with the increase of graphene size, the WF decreases. Moreover, the WF of zigzag graphenes is always bigger than that of armchair graphenes. We suggest that this phenomenon is caused by the crystal structure of graphene. Actually, crystal orientation has a big impact on the materials’ performance. The crystal structure of zigzag graphene is a cube structure, while the crystal structure of armchair graphene is a diamond structure. By comparing the WF between Fig. 2a, b, the WF of the graphenes (the graphene in Fig. 2a) with the unequal width and length would be bigger than that of the graphene (the graphene in Fig. 2b) with the equal width and length. The reduction gradient of the WF in Fig. 1a is also larger. Furthermore, the WF difference between the 6 × 6 and 7 × 7 supercells in the armchair and zigzag graphenes is small; we believe that the WF will be stable when the graphene size is up to the 6 × 6 supercells.

The band gaps of graphenes with various widths were also analyzed, as listed in Table 1. In general, graphenes with a small size will have a small band gap. However, as the width increases, the band gap decreased or even closed [44]. Son et al. have shown that graphene nanoribbons with homogeneous armchair- or zigzag-shaped edges all have energy gaps which decrease as the widths of the system increase [45]. Table 1 also shows that the band gap decreased with the size of graphene. Overall, the band gap of armchair graphenes is smaller than that of zigzag graphenes. Graphenes with the unequal width and length also possess a bigger band gap than graphenes with the equal width and length.

**Table 1:** The band gap of zigzag and armchair graphenes in various widths

Graphene size	Band gap (eV)		Graphene size	Band gap (eV)	
	Zigzag	Armchair		Zigzag	Armchair
1 × 7	0.696	0.540	–	–	–
2 × 7	0.351	0.311	2 × 2	0.345	0.334
3 × 7	0.145	0.123	3 × 3	0.129	0.007
4 × 7	0.054	–	4 × 4	0.009	0.003
5 × 7	0.024	–	5 × 5	0.009	–
6 × 7	0.004	–	6 × 6	–	–
7 × 7	–	–	7 × 7	–	–

### Effects of hydroxyls, defects, and positions of hydroxyls and defects on the WF

**Fig. 3:**
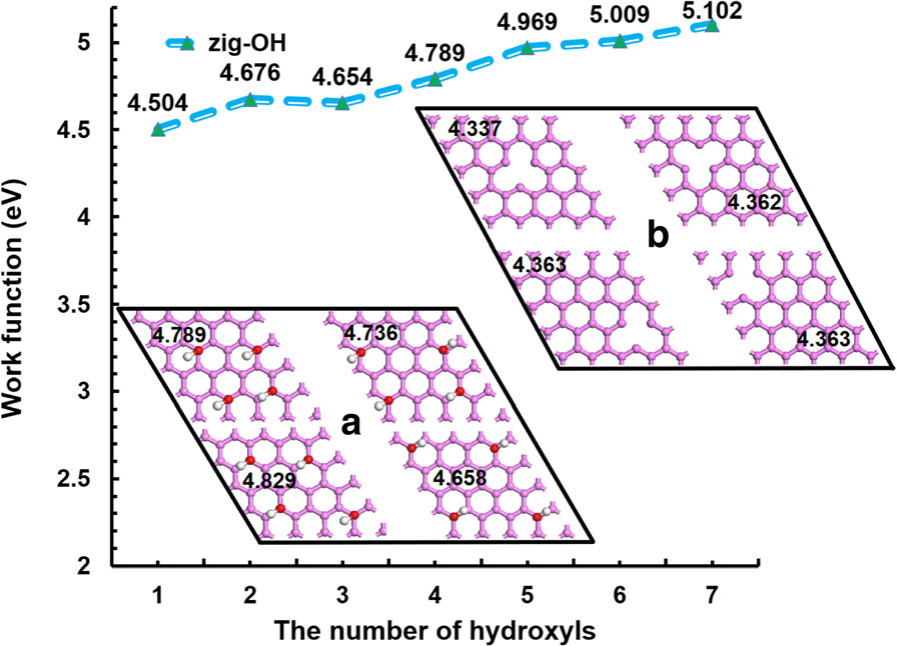
The relationship between the WF and the number of hydroxyls. The relationship between the WF and the number of hydroxyls; the size of graphene is set at 4 × 4 supercells. The inset pictures present four different distribution modes of hydroxyls (**a**) and defects (**b**)

Functionalization is always taken as a modification method in designing and improving the performance of the target material; hydroxylation is one of these methods. The influence of quantity and the position of hydroxyls and defects on the WF are analyzed, as shown in Fig. 3. Insets (a) and (b) illustrate the structure diagrams of hydroxyl and defect positions in graphene, respectively. In this calculation, pristine zigzag graphenes with 4 × 4-supercell size is selected, and the calculated WF is 4.479 eV, which is slightly smaller than that of the experiment result [12]. The hydroxyl modification will result in WF increase. Kang et al. determined the WF value of oxide graphene through experiment was 4.91 eV [27]. However, the number of the functional groups and their positions were not reported. The WF of zigzag graphenes with one hydroxyl we calculated is 4.504 eV, which is bigger than that of pristine zigzag graphenes. Along with the increase of the hydroxyls, the WF increases. Moreover, the increment is relatively large; the maximum WF reaches 5.102 eV. This result is due to the hydroxyl effect, which is highlighted with the increasing number of the hydroxyls. In addition, four hydroxyls are chosen to analyze the effect of the distribution of functional groups on the WF. Inset (a) gives four different ways hydroxyls can be distributed; the distributions are symmetrical. With intensive distribution, the WF is large. However, with dispersed distribution, the WF is small. The maximum value of WF is 4.829 eV, whereas the minimum value of WF is 4.658 eV. This phenomenon should be caused by the aggregation effect of hydroxyls. In addition, four different defect sites in the 4 × 4 graphene are investigated, as shown in inset (b). In general, the defects will result in the decrease of graphene WF. Bae et al. showed that the graphene WF was smaller when the vacancy existed. And the smaller the defect ratio was, the smaller the WF became [46]. The WF of graphene with the defect at the center is 4.337 eV, whereas the WF of graphene with the defect deviated from the center is larger at 4.363 eV, which is slightly smaller than that of the 4 × 4 pristine zigzag graphene. This difference means that the defects in the center have more impact on the structure, so the WF is at its smallest. Therefore, we suggest that the central defect sites have a large effect on the WF, whereas defects deviated from the center have a smaller effect. Kim et al. found that hole doping leads to a difference in the WF by as much as 400 meV, which is consistent with what we are computing [47].

### Effect of the dopants of B, N, Al, Si, and P on the WF

**Fig. 4:**
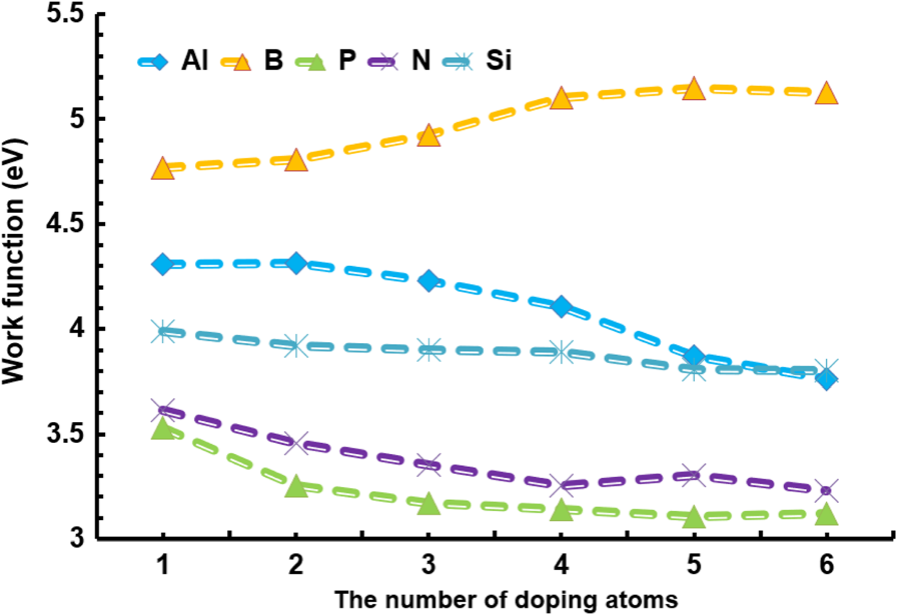
The relationship between the WF and the number of doping atoms. The relationship between the WF and the number of doping atoms. Different types of dopants, e.g., Al, B, P, N, and Si, are doped in the graphene with the size of 4 × 4 cells

Doping is an effective way to control the WF, band gap, and adsorption properties. Thus, the doping effects and concentrations are investigated in this study. Figure 4 shows the effect of different dopants on the WF; the graphene’s size is 4 × 4 supercells. Dopants from 1, 2, 3, 4, 5, and 6 atoms respond to the concentrations at 2.4, 4.9, 7.3, 9.8, 12.2, and 14.6%, respectively. The effect of dopants on the WF is significant and follows a certain trend. First, the WF of all the doped graphenes decreases as the concentration increases except for the B-doped graphene, which displays an opposite effect. Legesse et al. also found that the WF of the alkali metal-doped graphene decreases with the increase of the concentration [48]. Second, the increment of the WF in B- and Al-doped graphene is relatively bigger than that of the other graphenes. By comparing the WF value, p-type graphenes doped with B and Al have bigger WF, and the maximum value is up to 5.148 eV for B-doped graphene at a concentration of 14.6%. By contrast, the WF is much smaller in n-type graphenes doped with N and P; the minimum value is decreased to 3.23 eV at a concentration of 14.6% in P-doped graphene. Kwon et al. also showed that the p-dopants would increase graphene WF from 4.2 to 5.14 eV [49]. Kvashnin et al. also demonstrated the phenomena that B doping would cause WF increase, but N and P doping resulted in WF decreasing [19, 50]. In addition, the WF of Si-doped graphenes is relatively stable. This may be due to the fact that C and Si atoms are congeners. Therefore, we suggest that the p-type doping would lead to WF increasing; however, the amount is determined by the doping elements. The WF tends to be stable at the concentration of 14.6%. On the other hand, n-type doping will make the WF decrease sharply.

Although the influence of dopants on the WF has been analyzed and has the vital significance for the graphene application, the feasibility of doping for various atoms is different. Thus, we calculate the formation energy of different doping atoms in GNRs. The formation energy [51] is described as Eq. (4):

4 $$ {E}_{\mathrm{formation}}={E}_{\left(\mathrm{GNRs}+d\right)}+{nE}_{\mathrm{C}}-{E}_{\left(\mathrm{GNRs}\right)}-{nE}_{\mathrm{d}} $$

where *E*_formation_ is the formation energy, *E*_(GNRs)_ is the energy of pristine GNRs, *E*_(GNRs **+** *d*)_ is the energy of doped GNRs, *d* is the doping atom, *n* is the number, and *E*_C_ and *E*_d_ are the chemical potentials determined for carbon and doping atoms.

**Fig. 5:**
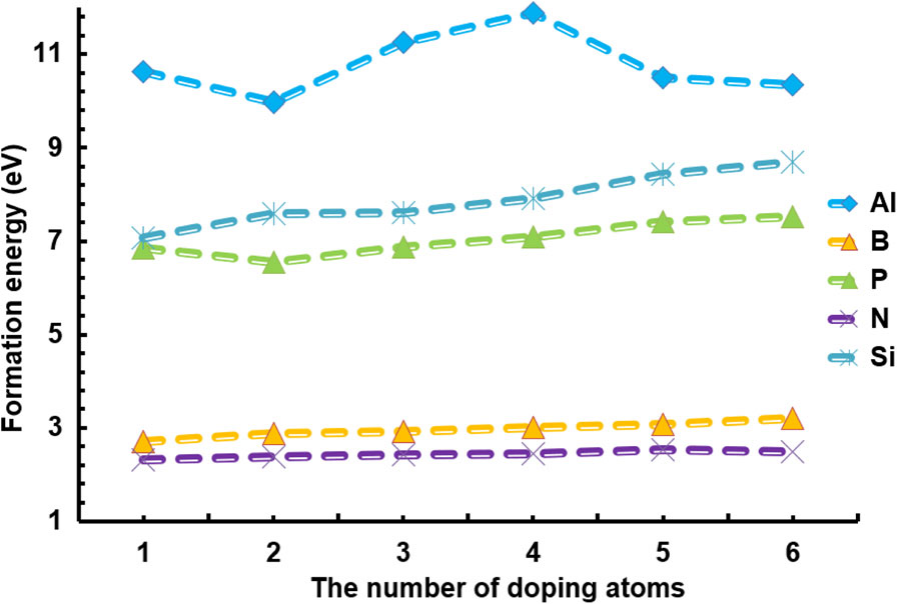
The relationship between the formation energy and the number of doping atoms. The relationship between the formation energy and the number of doping atoms. Different types of doping atoms, e.g., Al, B, P, N, and Si, are doped in the graphene with 4 × 4-cell sizes

The formation energy can be used to evaluate whether the feasibility of using atoms for doping is good or not. The smaller the formation energy is, the easier the doping becomes. Figure 5 shows that the graphene doped with Al has the largest but most unstable formation energy; the increase of the Al atoms leads to the dramatic changes of the structure in the graphene with 4 × 4-cell size. By contrast, the formation energy of B and N is very small, but small changes are evident with the increase of the number of atoms. The atoms of Al, Si, and P have more fluctuations in formation energy compared to the atoms of B and N. This is because the formation energies of Al, Si, and P in graphene are large, which means that the Al-, Si- and P-doped graphenes are less stable, especially the Al-doped graphene has the most unstable structure. They are relatively hard to be doped in graphene. Overall, the Al doping in graphene is difficult, whereas B and N doping are easier. The WF and formation energy of these dopants in graphene are recorded in Table 2.

**Table 2:** The WF and formation energy of the 4 × 4 graphene doped with the atoms of Al, B, P, N and Si, respectively

The number of doping atoms	WF (eV)					Formation energy (eV)				
	Al	B	P	N	Si	Al	B	P	N	Si
1	4.309	4.767	3.53	3.615	3.987	10.618	2.7	6.85	2.312	7.069
2	4.312	4.806	3.253	3.455	3.919	9.972	2.881	6.546	2.384	7.588
3	4.229	4.926	3.169	3.355	3.902	11.257	2.915	6.868	2.43	7.602
4	4.106	5.103	3.139	3.254	3.889	11.88	3.007	7.09	2.446	7.92
5	3.869	5.126	3.108	3.302	3.806	10.5	3.075	7.419	2.521	8.429
6	3.763	5.148	3.123	3.227	3.803	10.347	3.213	7.519	2.483	8.686

## Conclusions

The WF of graphene in different states, such as surface modification, doping, and defects, are investigated in this study. Basically, the WF decreases as graphene width increases. For the hydroxyl modification, the WF is large when the number of hydroxyls increases. Furthermore, when the distribution of hydroxyls is intensive, the WF is also increased. The defect would decrease the graphene WF, which does not depend on the positions. The p-type doping with B and Al would lead the WF to increase; however, the increased amount is determined by the dopants. The n-type doping with N and P reduces WF greatly. These discoveries will provide a theoretical support in controlling graphene and further improving the design of graphene-based devices.
